# Medical Legislation at Albany

**Published:** 1904-05

**Authors:** Frank Van Fleet

**Affiliations:** Chairman of the Committee on Legislation of the Medical Society of the State of New York, 60 East 77th St., New York


### Medical Legislation at Albany.

THE OPTICIANS AND OSTEOPATHS DEFEATED.—LETTER FEOM DR. VAN FLEET.

*Editor Buffalo Medical Journal*:

Sir—The legislature has adjourned, and both the optometry bill and the osteopathy bill have failed to become laws. There never was an effort made to secure legal recognition by any of these peculiar practitioners which had in it anything like the force of the opticians' bill of this year. In the beginning of the session the reports which I received from various quarters, seemed to indicate that the measure would become a law. But thanks to the support given the committee on legislation by the medical profession in all parts of the state, what seemed to be defeat for us has been turned into victory. I have no doubt that the medical profession can, through its representatives, control legislation affecting the medical laws, and the public health generally. In my experience I have found the various members of the legislature, not only willing, but anxious to learn the views of our profession, and the majority of them are willing to be guided by our advice.

I want to thank the medical profession, through your JOURNAL, for the very great aid given our committee, and to express the hope that sometime in the future when the necessity again arises, we may feel at liberty to call on you and the united profession for the same kind of aid.

FRANK VAN FLEET,
*Chairman of the Committee on Legislation of the Medical Society of the State of New York,*
60 East 77th St., New York.

April 16, 1904.

